# Pathogenesis and Mechanisms of SARS-CoV-2 Infection in the Intestine, Liver, and Pancreas

**DOI:** 10.3390/cells12020262

**Published:** 2023-01-09

**Authors:** Zaid Khreefa, Mallory T. Barbier, Ali Riza Koksal, Gordon Love, Luis Del Valle

**Affiliations:** 1 Department of Pathology, School of Medicine, Louisiana State University Health School of Medicine, New Orleans, LA 70112, USA; 2 Louisiana Cancer Research Center, Louisiana State University Health Sciences Center, New Orleans, LA 70112, USA; 3 Department of Pathology and Laboratory Medicine, Tulane University School of Medicine, New Orleans, LA 70112, USA

**Keywords:** SARS-CoV-2, COVID-19, ACE-2, gastrointestinal tract, intestine, liver, pancreas, autophagy, cytokine storm, MDSCs, inflammation

## Abstract

The novel coronavirus, SARS-CoV-2, rapidly spread worldwide, causing an ongoing global pandemic. While the respiratory system is the most common site of infection, a significant number of reported cases indicate gastrointestinal (GI) involvement. GI symptoms include anorexia, abdominal pain, nausea, vomiting, and diarrhea. Although the mechanisms of GI pathogenesis are still being examined, viral components isolated from stool samples of infected patients suggest a potential fecal–oral transmission route. In addition, viral RNA has been detected in blood samples of infected patients, making hematologic dissemination of the virus a proposed route for GI involvement. Angiotensin-converting enzyme 2 (ACE2) receptors serve as the cellular entry mechanism for the virus, and these receptors are particularly abundant throughout the GI tract, making the intestine, liver, and pancreas potential extrapulmonary sites for infection and reservoirs sites for developing mutations and new variants that contribute to the uncontrolled spread of the disease and resistance to treatments. This transmission mechanism and the dysregulation of the immune system play a significant role in the profound inflammatory and coagulative cascades that contribute to the increased severity and risk of death in several COVID-19 patients. This article reviews various potential mechanisms of gastrointestinal, liver, and pancreatic injury.

## 1. Introduction

Coronavirus disease-2019 (COVID-19) was first reported in Wuhan, China, in December 2019, after which it rapidly spread, causing a significant global public health problem. At the time of writing this review, there have been over 632,953,782 confirmed cases and 6,593,715 deaths worldwide; the United States appears to be the most affected country, with 96,752,266 cases reported, albeit this may be due to the lack of proper testing and reporting in other regions [1]. This disease is caused by severe acute respiratory syndrome coronavirus 2 (SARS-CoV-2), a single-stranded and enveloped positive RNA coronavirus belonging to the genus *Betacoronavirus* [2]. This novel virus has a 29.9 kB genome composed of 14 open frames that encode 29 proteins; of these, 16 are non-structural proteins (nsps), 4 are structural proteins, including the nucleocapsid, spike glycoprotein, envelope, and membrane glycoprotein, and the remaining 9 are accessory proteins [3,4,5]. The spike glycoprotein contains a receptor binding domain that enables the virus to bind to human angiotensin-converting enzyme 2 (ACE2) receptors and infect host cells [6,7,8]. Spike protein mutations result in the emergence of new viral variants, which can be more contagious and potentially resistant to vaccines [9,10].

Initially, SARS-CoV-2 infects the upper respiratory tract epithelium, where respiratory epithelial cells containing ACE2 receptors bind with the virally encoded spike glycoprotein. Transmembrane protease serine 2 (TMPRSS2) receptors prime the spike protein and promote viral endocytosis [7]. After primary infection, peak viral shedding occurs during the first week of infection and likely contributes to the high transmissibility of the virus [11]. The severity of COVID-19 ranges from asymptomatic to critical, with more than 80% of cases classified as a self-limiting mild-to-moderate disease presenting with symptoms that may include fever, headache, anosmia, and mild cough [12,13]. Up to 20% of patients will develop severe disease including approximately 5% classified as critical [14], where widespread infection of lung alveolar epithelial cells causes extreme disruption of gas exchange in the alveoli, contributing to the development of severe hypoxemia, edema, and lung fibrosis [9]. Viral dissemination to the lower respiratory tract follows, resulting in the development of viral pneumonia [15], and some patients exhibited severe adverse outcomes complicated by acute respiratory distress syndrome, respiratory failure, and in 1–2% of cases in the US, death [16,17].

Intracellularly, the viral genome is uncoated, released, and translated into various viral components. The replicase gene involves two open reading frames (ORFs) that encode the viral sections of the replication–transcription complex (RTC) [18,19]. Expression of the replicase gene and subsequent encoding of ORF1a and ORF1b leads to the production of replicase polyproteins, pp1a and pp1ab, respectively. The replicase polyproteins are cleaved by viral proteases into 11 (pp1a) or 16 (pp1ab) individual subunits of the RTC that are capable of associating with each other and host cell components to induce proviral changes in the environment. Nsp3, nsp4, and nsp6 have been shown to induce a change in membrane formation resulting in virus-induced double-membrane vesicles (DMVs) [19], most likely derived from the endoplasmic reticulum (ER) [20]. The RTC participates in the replication and subsequent generation of a nested set of subgenomic RNA, which is translated into viral structural proteins (S, M, N, and E). The newly formed virus particles are assembled by exploiting the membranes and other elements of the ER and Golgi complex. Eventually, the mature virions are transported in vesicles and released from the cell via exocytosis into the extracellular compartments [21,22].

Viral modifications in the autophagic and extracellular vesicle pathways contribute to the overactivated and impaired immune responses that ultimately promote viral replication, the release of viral particles into the extracellular compartment [21], and the hypercoagulative states related to disease severity. Research shows that several cell types are affected by SARS-CoV-2 infection [23,24,25], with some having deleterious effects on disease severity and outcome, including epithelial, endothelial, and immune cells. Various immune and inflammatory changes induced by the virus contribute to endothelial cell damage and dysfunction in COVID-19 that may induce hypercoagulative states [26,27,28]. In hypercoagulative states, there is an increased chance of developing lung thrombi and emboli that contribute to the respiratory distress and mortality encountered in COVID-19 patients.

The high expression of ACE2 in the alimentary tract makes the small and large bowels vulnerable to SARS-CoV-2 infection [29,30]. Although fever, cough, and shortness of breath are the most common symptoms, around 17.6% of the patients present with gastrointestinal manifestations [31]. Of the 17.6%, anorexia is the most common presentation in 26.8% of cases, followed by diarrhea (12.5%), nausea and vomiting (10.2%), and abdominal pain (9.2%). Furthermore, liver injury is observed in 15% to 53% of the cases. The real-time reverse-transcription polymerase chain reaction (RT-PCR) assay is the standard test for laboratory diagnosis of SARS-CoV-2 samples collected from infected patients, including stool samples [32]. This review summarizes the pathogenesis and potential mechanisms of gastrointestinal, liver, and pancreatic injuries in patients with COVID-19.

## 2. Mechanisms of SARS-CoV-2 Infection in the Intestine

### 2.1. Viral Kinetics and Physiologic Luminal Barriers

The positive detection of SARS-CoV-2 in stool constitutes evidence that the virus exists and replicates in the intestines of infected patients [33,34,35]. More than half of the hospitalized patients with COVID-19 demonstrated positive stool samples, particularly those with diarrhea. Interestingly, some studies have shown that a subset of COVID-19 patients still presents with positive SARS-CoV-2 stool after negative nasopharyngeal tests [36,37]. The exact mechanism for how the virus infects the gastrointestinal tract is unknown. Still, hypotheses include fecal–oral transmission, direct digestion after viral exposure, and mucociliary clearance of respiratory virions that may allow entry into the alimentary tract via the esophagus [38]. SARS-CoV-2 viral shedding has been detected in the stool of COVID-19 patients and, as with most enteric viruses, it could be a potential fecal–oral transmission route [34,39,40,41,42,43]. A study showed that SARS-CoV-2 was detected on toilet surfaces and the bathroom door handle in a patient room with confirmed fecal viral positivity by RT-PCR [44]. For this reason, individuals using poorly sanitized areas, such as public toilets, are potentially more vulnerable to viral transmission after touching the nose, mouth, or eyes with contaminated hands. Furthermore, SARS-CoV-2 has been detected in raw urban sewage as a part of population-wide surveillance and may be particularly beneficial as an early warning system in monitoring for mutated variants [45,46]. The virus’s genetic diversity due to high mutation rates threatens COVID-19 treatment options and the current vaccination programs. A drawback to wastewater surveillance is that samples may reveal artificial genomes that do not correspond to a virus circulating in the population; however, they can still be linked to specific variant clusters and provide information about variants circulating in an area.

Enteric RNA viruses, including rotavirus, norovirus, and astrovirus, are mainly transmitted via the fecal–oral route [47,48,49]. After ingestion, these viruses are carried to the intestine, where they invade the epithelial cells, cause gastrointestinal symptoms, and are excreted with stool. SARS-CoV-2 may exhibit a similar mechanism of transmission and pathogenesis in the gastrointestinal tract [30,39,43,50,51]. Most enveloped viruses would not survive in the harsh alimentary tract conditions; however, a notable exception is enteric coronaviruses. Though the survival mechanisms are unclear, some studies suggest the possibility of SARS-CoV-2 surviving and being transported to the intestines in saliva or secretions [30,52,53]. Gastric acid is an essential physiological barrier that plays a vital role in the inactivation of swallowed pathogens. Studies have shown that the use of a proton pump inhibitor, a medication that causes a reduction in gastric acid production, has been linked with increased mortality in patients with COVID-19 infection [54,55,56,57]. The suppression of gastric acid can facilitate the passage of the virus through the stomach to the intestine [58]. Because of the drug-induced, less acidic, virus-friendly environment in the intestine and the high viral load in the stool, the virus may survive for a longer time in the gastrointestinal tract compared to the respiratory tract [43].

### 2.2. Possible Viral Entry Mechanisms to the Intestinal Epithelial Cells

**Figure 1:**
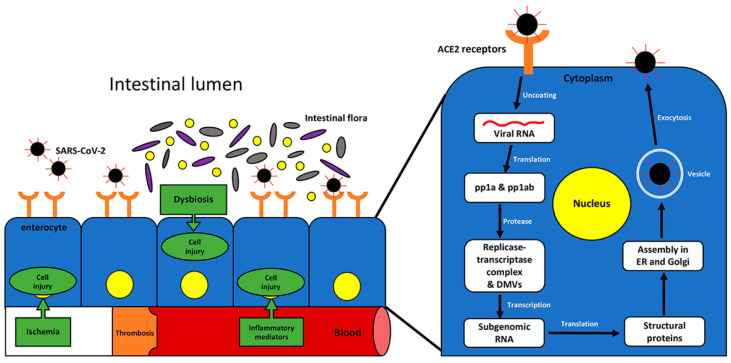
**Mechanisms of infection and pathogenesis of COVID-19 in the intestine.** The molecular pathogenesis of SARS-CoV-2 infection of epithelial gastrointestinal cells is depicted, including the viral replication cycle.

ACE2 is an important receptor on the cell membrane of host cells. It is widely distributed in various organs, including the nasopharynx, lung, esophagus, stomach, intestine, liver, and pancreas. Studies have shown that ACE2 is abundantly expressed in the gastrointestinal tract, particularly the colon, at approximately 100 times more than in the lung [59,60,61]. Multiple proteins are anchored on the viral surface, including structural spike, membrane, nucleocapsid, and envelope proteins. The interaction between the virus’s transmembrane spike glycoprotein and the ACE2 receptor on enterocytes promotes the invasion of host brush border cells. The spike protein must be primed by TMPRSS2 which promotes endocytosis and facilitates the invasion of intestinal enterocytes by the SARS-CoV-2 virus [62]. Enterocytes are intestinal absorptive cells that create the brush border of the luminal surface of the intestines. These cells highly express ACE2 and TMPRSS2 receptors [7,62,63]. The brush border has interspersed immune cells called microfold cells (M cells) that initiate localized immune responses by taking up antigens from the lumen of the intestines and delivering them to antigen-presenting cells and other immune cells that migrate to a pocket on the basement membrane surface of M cells. However, in the presence of some viruses, the ingested viral particles are not transported across the M cell to underlying lymphoid follicles but instead hijack the host cells’ machinery and begin actively replicating within the M cells, like the well-studied coronavirus transmissible gastroenteritis virus [64,65]. The abundant viral replication results in destroyed M cells, leading to mucosal inflammation and diarrhea. As M cells are destroyed, SARS-CoV-2 avoids immune detection via antigen presentation to the underlying lymphoid follicles. This is carried out by apical virion exocytosis and by utilizing spike protein fragments, which remain in the cytosol after replication and virion assembly, to invade adjacent uninfected cells basolaterally [66]. Since a highly weakened immune response may be launched from the direct infection of enterocytes by inducing the release of cytokines, viral particles in the lumen can continue to infect uninfected enterocytes, allowing viral proliferation and subsequent damage to the gastrointestinal tract. Figure 1 depicts the entry and viral life cycle mechanisms in intestinal epithelial cells.

### 2.3. Autophagy and the Replication of SARS-CoV-2

An essential part of the antiviral defense mechanism for cells is autophagy. Autophagy is crucial to maintain cellular homeostasis, remove misfolded proteins, eliminate viral particles, and reduce virion formation [67]. Cells conduct this process in response to cellular stress by using autophagy machinery to deliver materials in the cytosol to the interior of lysosomes for degradation, subsequently recovering metabolic energy sources and targeting damaged or unnecessary organelles to lysosomes for removal. However, autophagy pathways and machinery can be hijacked by certain invading viruses, including SARS-CoV-2, and then used against host cells to aid viral replication, proliferation, and translocation.

The incorporation of viral genes into the host cell genome can alter the expression of autophagy-related genes via serum starvation, pH imbalances, stimulation of T and B cell antigen receptors, and cytokine stimulation. In studies involving HCV, an RNA virus whose viral replication kinetics are similar to SARS-CoV-2, it has been shown that the virus activates autophagy during the acute infection period [68,69]. Several replication intermediates are synthesized in the acute period of the COVID-19 infection. The clearance of intermediate replication products by virus-activated autophagy has been suggested as the mechanism of escape from interferon (IFN) lambda-related immune response [70]. Like other coronaviruses, the formation of pseudo-enveloped vesicles during SARS-CoV-2-induced autophagy relies on ER-derived membranes [71]. These viral-induced DMV membranes lack properly functioning ER chaperones and protein markers typically found in uninfected ER-derived membranes that assist the autophagic machinery with substrate recognition and targeting to lysosomes [67,72,73].

LC3 is a key protein in the autophagy pathway and is involved in substrate selection and the formation of autophagosomes [74,75]. Autophagy induction can be detected by converting LC3B-I, a widely distributed cytosolic protein, to LC3B-II, a conjugated form of LC3B-I concentrated in autophagolysosomal membranes [75,76]. SARS-CoV-2 infected cells contain the viral protein nsp15, which is a uridine-specific endoribonuclease unique to the order *Nidovirales*, that processes mature viral RNA during replication, and degrades viral sequences that activate host immune sensors by functioning as an RNase and degrading viral dsRNA intermediates, thus preventing immune recognition and delaying type I IFN response [77,78]. In a significant viral load, nsp15 overexpression has been shown to decrease LC3B-II and lead to the accumulation of p62, an autophagic receptor, indicating that this viral protein is involved in blocking autophagy induction, although the precise mechanisms are not clear [78,79,80]. Nsp15 has been considered a target for therapeutic techniques due to studies conducted that revealed that loss of nsp15 function correlates to both decreased replication and pathogenesis [81,82].

Studies show that SARS-CoV-2 inhibits autophagic flux using viral proteins ORF3a and ORF7a by disturbing the interplay between autophagosomes and lysosomes [83]. ORF3a plays a role in inducing autophagy [84]; however, it induces incomplete autophagy by interacting with and deregulating Beclin 1, an essential autophagy protein that regulates downstream autophagosome formation and maturation, and subsequently causes a buildup of immature autophagosomes incapable of phagolysosome fusion [85]. This prevents autophagic cargo degradation and aids in promoting viral RNA replication [86]. ORF7a was found to disrupt and reduce lysosomal acidity, which prevented autophagosomal degradation [78,83]. This leads to the accumulation of lysosomes that are incapable of autophagosomal degradation. Additionally, ORF7a may promote virion release by avoiding degradation [83]. ORF8 is a SARS-CoV-2 open reading frame that encodes a viral protein detected in LC3-labeled autophagosomes [87]. It was shown that this viral protein directly interacts with MHC I leading to MHC I downregulation and impairment in viral antigen presentation, which protects infected cells from being recognized and eradicated by T cells. LC3 usually facilitates lysosomal fusion and degradation, but studies [88,89] have revealed that viral protein disruption impairs LC3 function and allows SARS-CoV-2 virions to use the lysosomal trafficking pathway as a method of release from infected cells.

IRF8 is a transcription factor that promotes macrophage and dendritic cell differentiation and is critical for the expression of autophagy-related genes, stress-induced autophagy regulation, antigen presentation, and clearance of intracellular pathogens [90,91]. Studies show that COVID-19 patients have significantly reduced expression of IRF8, which contributes to the impairment of immune responses mediated by autophagy pathways [89,92,93] and ultimately leads to increased viral proliferation and deregulated inflammatory pathways due to IRF8′s role in MDSC pathology, which is discussed below.

Autophagy flux impairments can lead to ER stress, causing a subsequent increase in unfolded proteins leading to chronic tissue injury and excessive reactive oxygen species (ROS) production due to mitochondrial dysfunction [79,94]. Under normal conditions, autophagy suppresses inflammatory factors, but defective autophagy seen in SARS-CoV-2 reveals that the virus and viral-altered processes may uncontrollably stimulate inflammatory pathways [67,79,95]. One example is that ROS accumulation stimulates various mechanisms of inflammation, including inflammasomes and calpain, to produce IL-1B and IL-1a, respectively [96,97,98]. They also increase RIG-I-like receptors to enhance type-I IFN production. Inflammation in the gastrointestinal system can lead to diarrhea and severe abdominal pain. Autophagy is normally a major defense mechanism used to regulate and suppress levels of proinflammatory cytokines by efficiently removing stimuli, such as defective mitochondria, or pathogens, such as viral components, which activate these inflammatory factors. Dysregulation of autophagy-related genes, such as Beclin 1 and LC3 as seen in SARS-CoV-2 infection, causes a marked increase in cytokine secretion [96,99,100], implicating autophagy dysfunction as a significant contributor to an uncontrolled cytokine storm that is accountable for COVID-19 severity and death. The cytokine storm in turn will cause severe intestinal symptomatology, including diarrhea. In addition, the reduced activation of mTOR, a set of kinase-type proteins directly involved in autophagy induction, results in reduced secretion of antimicrobial agents by Paneth cells allowing uncontrolled viral proliferation in the intestines [67,92,101,102].

### 2.4. The Role of Extracellular Vesicles/Exosomes in Viral Transmission and Spreading to the GI System

SARS-CoV-2 RNA has been detected in blood samples of infected patients [42,103,104], making the hematologic dissemination of the virus to the digestive system another proposed alternative route of transmission. Extracellular vesicles (EV) are found in various biofluids and serve as carriers that transfer biomolecules to target cells all over the body. EV secretion is a known pathway aiding in the transmission and spread of several RNA viruses using exosomes, the smallest EV subclass; however, the mechanisms that viruses use to accomplish this is still up for debate [105,106,107]. One model proposes that the autophagic pathway is involved as LC3-labeled “autophagosome-like” organelles selectively capture a cluster of viral particles and release them non-cytolytically from an infected cell [108,109,110]. These released particles can subsequently be picked up by exosomes in biofluids and transported to distant organs. An additional proposed mechanism states viral proteins, RNA, or entire virions may be directed into exosomes that are then expelled from the infected cell via exocytosis into the extracellular space [107,111]. Exosomes then bind to target cells and induce susceptibility for viral infection within these healthy cells using membrane receptors, such as ACE2 and CD9, while fusing to the target cell membrane and unloading viral cargo [105,111,112]. It is thought that EVs play an important role in the transformation of COVID-19 infection from a local to a systemic infection contributing to the development of systemic inflammatory response syndrome [113]. This is a potential mechanism demonstrating how SARS-CoV-2 proteins, RNA, and even entire virions may be transported from the respiratory system after primary infection through biofluids and into the GI system to infect intestinal cells and cause GI illness. In a recently published article, it has been shown that inflammatory signals are carried to cells in distant organs through Tenascin C and Fibrinogen Beta carried by exosomes obtained from COVID-19 patients [113]. In another study, it was shown that some of the drugs that inhibit exosomes through different mechanisms inhibit SARS-CoV-2 replication [114]. All these preliminary results show that extracellular vesicles and exosomes have an important role in GI manifestations caused by direct or indirect mechanisms of COVID 19 infection.

### 2.5. Proinflammatory Effects of COVID-19 Infection and Systemic Inflammatory Response Syndrome

The production and release of large quantities of proinflammatory cytokines are referred to as a “cytokine storm” and have been well-established in the COVID-19 outbreak [115,116,117,118]. It is attributed to a systemic inflammatory reaction and multiple organ dysfunction, including the gastrointestinal system, seen in more severe cases [30,93,119]. Viral infections of intestinal cells cause intestinal epithelial cellular stress and have been implicated in inducing the release of various proinflammatory cytokines and chemokines that normally work to limit viral infection and spread [120,121]. In the presence of SARS-CoV-2, these chemical immune responses result in tissue and organ damage, coagulopathy, and acute respiratory distress syndrome (ARDS), which in turn increase the release of even more proinflammatory cytokines and chemokines, creating a cycle of uncontrolled inflammation and damage [122,123,124].

Various mechanisms have been explored regarding the molecular pathogenesis of SARS-CoV-2 in the intestines. ACE2 plays a role in controlling the absorption of amino acids, including tryptophan, in the small intestine [125]. Tryptophan is involved in regulating the suppression of intestinal inflammation and modulating intestinal microbiota. Infected enterocytes result in altered ACE2 expression, causing impaired tryptophan absorption and subsequent deregulation of tryptophan metabolism [126]. Alterations to tryptophan metabolism deregulate the metabolic ability to suppress intestinal inflammation [125,127]. Another mechanism involved in the pathogenesis of SARS-CoV-2 in intestinal cells involves the significant induction of type-I and type-III IFNs and the significant upregulation of proinflammatory mediators, indicating that the virus massively triggers innate immune responses [128].

Some of the viral nsps, such as nsp14, can block IFN-I responses and antagonize cytokine production by inducing host mRNA degradation and inhibiting the phosphorylation of both signal transducer and activator of transcription (STAT) 1 and interferon regulatory factor 3 (IRF3) [78,129,130,131]. In healthy cells, IRF3 plays a role in the innate antiviral response by inducing type-I IFNs. Nsp15 can inhibit IRF3 translocation by antagonizing IFN production [77], and nsp15-mediated inhibition of IRF3 is an additional method by which SARS-CoV-2 can evade the host’s immune response [132,133].

Infected cells release mediators and chemokines that quickly cause local neutrophil accumulation and the additional secretion of cytokines and chemokines, attracting further immune cells such as T lymphocytes [134]. The activation of T lymphocytes leads to a high expression of various cytokines, including IFN-γ, IL-2, IL-6, IL-7, IL-10, and human granulocyte-macrophage colony-stimulating factor [135,136]. These inflammatory cytokines can cause a unique immune activation, ultimately promoting host cell necrosis [50]. Microscopically, the epithelium of the digestive tract shows patchy plasma-lymphocytic infiltration with interstitial edema [137,138]. Vasculitis, active inflammation, viral inclusions, and submucosal fibrin thrombi have also been reported [139]. As a result, SARS-CoV-2 may cause gastrointestinal symptoms by the cytotoxic effect secondary to direct viral invasion in target cells and/or immune-mediated tissue and end-organ damage [140]. In severe cases, these immune responses fail to clear out the pathogens due to the impaired autophagy pathway mentioned above.

### 2.6. Myeloid Derived Supressor Cells (MDSCs)

An interesting finding in SARS-CoV-2’s mechanisms of immune evasion concerns MDSCs. MDSCs, or myeloid-derived suppressor cells, are pathologically activated monocytes and granulocytes [141]. These cells have unique metabolic and gene expression profiles that enable them to inhibit immune responses involving T cells, B cells, and natural killer (NK) cells and induce systemic and local immunosuppression while promoting the accumulation of other types of immunosuppressive cells, such as T-regulatory cells [142,143,144]. In the context of cancer, MDSC function worsens the disease, but in autoimmune and infectious diseases, MDSCs can limit severity [141]. Several studies have shown increased MDSCs, and more specifically, monocyte-MDSCs (M-MDSCs), in the blood samples of severe COVID-19 patients [92,145,146]. Multiple studies have demonstrated a significant expansion of M-MDSCs in SARS-CoV-2 infections that may occur via IRF8 and IL-6-mediated mechanisms [92,147,148,149]. IL-6 is a pleiotropic cytokine with both anti- and proinflammatory activities and was shown to be significantly increased in severe COVID-19 cases compared to mild cases [150]. This suggests that IL-6 can serve as a marker to indicate the transition from mild to severe COVID-19. High concentrations of IL-6 may be responsible for maintaining MDSC’s suppressive activity of T cells permitting prolonged viral survival. As mentioned earlier, IRF8, which was shown to be significantly reduced in COVID-19, promotes macrophage and dendritic cell differentiation [151] and has a critical role in autophagy, antigen presentation, and clearing intracellular pathogens [90]. Autophagy controls MHC II antigen presentation and is crucial for boosting tolerance and anti-inflammatory processes by myeloid cells [67,91,101,152]. Still, this process is impaired when IRF8 is downregulated, preventing immune cells from responding to antigens. IL-6 upregulation and IRF8 downregulation allow the maintenance of MDSC-induced immune suppression and subsequent advancement of hyperinflammation activation, contributing to the development of cytokine release syndrome or ‘cytokine storm.’

L-arginine is a semi-essential amino acid and intestinal L-arginine is involved in protein production, urea removal, and nitric oxide (NO), urea, creatine, and polyamine synthesis. It also plays a role in regulating intestinal pathogens to prevent infection. In healthy adults, adequate amounts of L-arginine are synthesized endogenously, but in the context of catabolic stress seen in COVID-19, endogenous synthesis is insufficient [145]. Depletion of free L-arginine has been implicated as a mechanism of T cell dysfunction [153,154,155,156]. This is most likely a result of the significant expansion of MDSCs seen in COVID-19 patients [145,157]. MDSCs highly express arginase I and iNOS that both metabolize free L-arginine, and one study demonstrated that L-arginine consumption by MDSCs induces platelet activation due to the reduced NO production secondary to the depletion of L-arginine. NO plays a role in inhibiting platelet activation [158,159], therefore an L-arginine deficiency is associated with increased severity and thrombotic complications reported in severe COVID-19 cases [145,160,161]. MDSCs also express specific ligands that interact with T cells and lead to T cell exhaustion, further suppressing the T cell response. Therefore, in response to viral invasion, the accumulation of MDSCs in the intestines inhibit the gut mucosal immune response allowing increased viral proliferation and subsequent inflammation.

### 2.7. The Effects of COVID-19 Infection on the Coagulation System in Gastrointestinal Tract

In critically ill patients, infectious complications are common and may activate multiple systemic inflammatory and coagulation responses that are intended to defend the host, but specific viral genes and proteins may manipulate the response leading to disseminated intravascular coagulation. Coagulopathy occurs when blood’s ability to form clots is impaired and has been seen in COVID-19 more frequently and with greater severity when compared to other diseases caused by respiratory viruses [162,163,164,165]. COVID-19-associated coagulopathy may present as either microthrombi or macrothrombi, and both can cause multiple organ damage, increased disease severity, and death [166]. Immunothrombosis occurs when viral pathogens and their components bind to pattern-recognizing receptors (PRRs) on immune cells and trigger inflammatory responses inducing proinflammatory cytokines that activate coagulation, particularly when damaged vascular endothelium, also known as vasculopathy, and resultant endothelial dysfunction promotes clot formation [27,28,167,168,169,170,171]. The pathogenesis of COVID-19 vasculopathy is still debated, with two prominent theories. The first is that the virus causes direct endothelial injury [6,60,172,173,174,175]; however, arguments have been made that endothelial cells do not express ACE2 or express it at very low levels, making this cell type resistant to infection [176,177]. The second is that the viral infection triggers a cytokine storm, the source of endothelial cell injury [177,178,179,180]. However, with disease progression, it is likely that multiple factors play into the severity of damage and subsequent thrombosis that causes organ damage. SARS-CoV-2 binds to ACE2 on lung epithelial cells resulting in a local ACE2 deficiency. ACE2 regulates the renin-angiotensin-aldosterone system (RAAS), which is involved in blood pressure and vascular resistance regulation, and the kallikrein-kinin system, which regulates vasoactive peptides [8,181]. Therefore, the deregulation of ACE2 can increase kinin activity and result in angioedema [166,182]. A ‘kinin storm’ may occur due to systemic kallikrein-kinin system activation resulting in increased inflammation, vascular permeability, effusion, and ultimately organ damage. Increased vasodilation and permeability allow vascular leakage of various components involved in the hyperactive innate immune response, such as autoantibodies, ROS, cytokines, and chemokines. These leaked substances cause a fibrinolysis imbalance contributing to glycocalyx damage and vascular endothelial injury that results in endothelial prothrombogenic activity and increased levels of von Willebrand factor, fibrin, tissue factor, and P-selectin. These increases contribute to the already overactivated coagulation cascade induced by catastrophic inflammation and increased cytokine production. This intense inflammatory state causes a severe hemostatic system derangement, systemic blood coagulation, and downregulation of the fibrinolytic and anticoagulant pathways, increasing the risk of thrombi formation. RAAS dysregulation is a major contributor to endothelial damage and is a significant component in microthrombi formation and COVID-19’s subsequent ischemic manifestations [183].

Ischemic colitis, a rare presentation of SARS-CoV-2 involvement in the colon, has been reported in some cases [138,183,184,185]. Microscopic examination of these cases showed coagulative mucosal necrosis with crypt apoptosis and small vessel thrombosis, mainly noted in the submucosal vasculature [184]. Reports indicate that patients with COVID-19 have high levels of D-dimer and fibrinogen, which increases the risk of micro- and macro-circulatory thrombosis [28,186,187]. Additionally, hypoxia that occurs in severe cases of COVID-19 can promote thrombosis formation by increasing the viscosity of the blood and activating the hypoxia-inducible transcription factor-dependent signaling pathway [171].

### 2.8. COVID-19 and Microbiota Changes

Studies of patients with GI-related COVID-19 symptoms lacked beneficial bacteria and showed a significant increase in opportunistic pathogens [188,189,190]. Anti-inflammatory bacteria normally found in healthy individuals were absent from the gut microbiomes of COVID-19 patients, with studies showing that the gastrointestinal virome functionality revealed increased inflammation, stress, and virulence responses and may be due to other viruses taking advantage of the host immune dysfunction [188]. As previously mentioned, ACE2 mediates the absorption of neutral amino acids, such as tyrosine, valine, and tryptophan in the intestine [8] and tryptophan plays a vital role in regulating gastrointestinal flora [126,127,191]. The binding of SARS-CoV-2 to ACE2 receptors in the bowel can reduce the number of free receptors and interfere with tryptophan absorption. This induces malabsorption and causes dysbiosis, an imbalance of the intestinal microbiota. Diarrhea seen in COVID-19 may result as a consequence of decreased ACE2 expression, subsequent reduced tryptophan absorption leading to gut dysbiosis, and dysregulated autophagic pathways. Downregulation of ACE2 in infected enterocytes induces intestinal dysbiosis. Dysbiosis inhibits mechanistic target of rapamycin (mTOR) which causes the activation of intestinal autophagy [101,192]. This causes autophagic degradation of NHE3, an intestinal epithelial brush-border Na+/H+ exchanger protein that regulates the NaCl absorption [193]. Reduced NHE3 levels reduce the intestine’s ability to absorb water which increases intestinal fluids and results in secretory-type diarrhea [194].

Studies have shown altered content of *Clostridium*, *Lactobacillus*, and *Bifidobacterium* in the intestinal flora of patients with SARS-CoV-2 [189,195,196,197]. A portion of intestinal flora are amino acid-fermenting bacteria that utilize arginine to function [198]. Alterations in intestinal flora may lead to an increased presence of L-arginine-fermenting bacteria leading to additional depletion of free L-arginine in the intestines. Depletion of intestinal L-arginine is known to increase MDSCs, suppress local T cell function as described above, as well as increase the production of ROS, thus exacerbating intestinal inflammation [145].

The dysbiosis of the bowel flora promotes viral replication and enhances the release of inflammatory proteins and cytokines, which ultimately contributes to the ‘cytokine storm’ state and subsequent widespread tissue damage, septic shock, and multi-organ failure [189]. Other factors altering gut flora in patients with COVID-19 include diabetes mellitus, old age, the use of antibiotics and antivirals, and steroids which can negatively affect the prognosis in these patients [199].

## 3. Mechanisms of SARS-CoV-2 Infection in the Liver

COVID-19-associated liver injury has been reported in several studies presenting with abnormal liver function biochemistries [200,201,202,203,204,205], and viral presence was confirmed by detecting SARS-CoV-2 in liver tissues [206,207,208,209]. In COVID-19, liver injury was characterized by elevated total bilirubin levels and increases in alanine transaminase and/or aspartate aminotransferases, and the level of these enzymes directly correlated with the severity of the disease [210,211,212,213].

**Figure 2:**
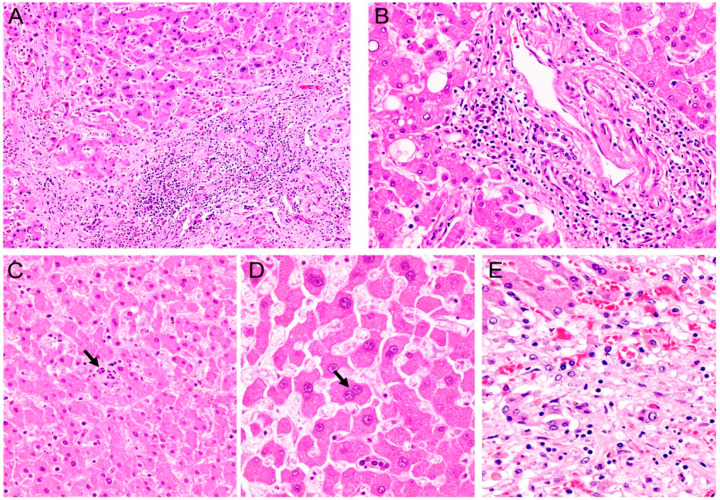
**Liver damage after SARS-CoV-2 infection.** Severe chronic portal inflammation is observed in livers of patients with acute COVID-19 (Panels (**A**,**B**), Hematoxylin and Eosin, original magnification 200× and 400×, respectively). At higher magnification the fragmented nuclei of apoptotic hepatocytes are present (Panel (**C**), arrow), as well as Binucleation of hepatocytes (Panel (**D**), arrow). In severe cases, extensive areas of necrosis were observed (Panel (**E**)) Panels (**C**–**E**), Hematoxylin & Eosin, original magnification 400×.

The exact mechanism for pathogenesis by SARS-CoV-2 of the liver is controversial and most likely involves not one, but several mechanisms. Several studies have reported the involvement of ACE2, the overproduction and release of inflammation mediators, coagulation and thrombi formation, and hypoxia [60,204,209,213,214,215,216,217,218]. ACE2 receptors were found to be expressed in cholangiocytes more than in hepatocytes [170]; however, histopathological analysis revealed abundant virus particles within the cytoplasm of hepatocytes [213]. This could indicate that the presence of SARS-CoV-2 initiates an upregulation of ACE2 in this cell subtype allowing for viral infection [214]. In addition to the presence of viral particles in the cytoplasm of hepatocytes, liver biopsies revealed prominent Kupffer cells, acidophilic bodies, portal tracts with chronic inflammatory infiltrates, and ballooning degeneration of hepatocytes with frequent mitoses. Ultrastructural examination showed mitochondrial swelling, endoplasmic reticulum dilation, and glycogen granule decrease, accompanied by massive hepatic apoptosis, which is a known mechanism utilized by cells to dispose of damaged or virally infected cells. The histopathological changes observed in the livers of patients with acute SARS-CoV-2 infection are depicted in Figure 2.

ACE2 is expressed 20 times higher in the biliary epithelial cells, also known as cholangiocytes, than in hepatocytes indicating liver injury may also contribute to bile duct injury [218]. Several studies also showed a decrease in CD4+ and CD8+ lymphocytes [219,220,221], which can be a direct result of the increased numbers of MDSCs, as discussed above.

**Figure 3:**
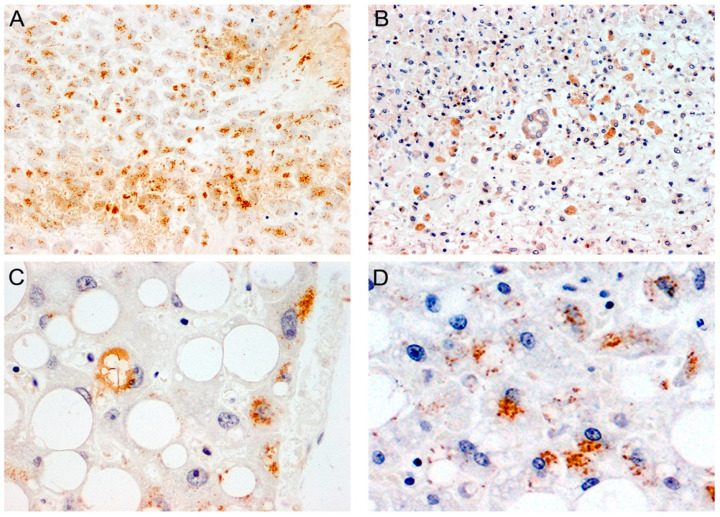
**Detection of SARS-CoV-2 proteins in liver samples.** Immunohistochemistry for SARS-CoV-2 Nucleocapsid shows robust immunoreactivity in hepatocytes, in areas of liver damage (Panel (**A**) original magnification 100× and Panel (**C**) original magnification 400×). Spike protein is also present in the cytoplasm of hepatocytes in areas of necrosis (Panel (**B**) original magnification 100×, and steatosis (Panel (**D**) original magnification 400×).

Additionally, KI-67 immunohistochemistry has shown an increased number of hepatocytes in the replicative phase with a proliferative index of 11.4% [206]. Prominent bi-nucleated hepatocytes with occasional apoptosis were also identified in some cases [213]. The findings of mitoses, apoptosis, and an elevated KI-67 index indicate potential cell cycle disruption caused by SARS-CoV-2 that warrants further investigation. Figure 2 demonstrates some of the histopathological features seen in the liver after infection with SARS-CoV-2. Figure 3 shows the immunohistochemical detection of viral nucleocapsid and spike proteins in hepatocytes of patients with active SARS-CoV-2 infection.

**Figure 4:**
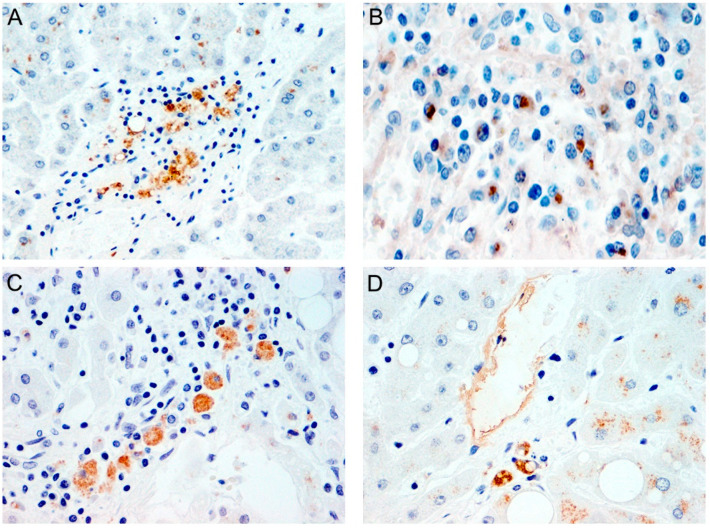
**Expression of SARS-CoV-2 proteins in liver inflammatory and endothelial cells from cases of acute fatal COVID-19.** Nucleocapsid is detected in portal inflammatory cells (Panel (**A**), original magnification 200×). Spike is also robustly expressed in portal lymphocytes (Panel (**B**), original magnification 600×). Nucleocapsid is also present in macrophages (Panel (**C**), original magnification 400×) and endothelial cells (Panel (**D**), original magnification 400×) in liver samples.

Liver endothelial cells were also found to abundantly express ACE2 [215] indicating that liver vascular injury induced by SARS-CoV-2 endothelial infection and replication may result in increased vascular permeability and damage allowing leakage of inflammatory cytokines and free ROS into liver tissues [222]. Interestingly, we found expression of both nucleocapsid and spike proteins in the endothelial cells of livers from patients with acute COVID-19 infections, indicating that the virus actively reaches the liver through the vascular system, either through endothelial cells of through macrophages or lymphocytes, which are also robustly positive for SARS-CoV-2 proteins. Endothelial damage exposes underlying collagen, which subsequently attracts platelets and neutrophils to promote NETosis and clot formation. Accumulation of platelets and immature neutrophils in the liver may further initiate endothelial damage and lead to the microvascular thrombi observed in severe and nonsurvivable cases of COVID-19 [170,223,224]. Figure 4 shows the expression of nucleocapsid and spike in portal inflammatory cells, lymphocytes, macrophages and endothelial cells.

Drug-induced liver damage is another contributing factor to hepatocyte injury in patients with COVID-19. Many medications used to treat hospitalized patients have a significant hepatotoxic effect, including acetaminophen, IFN, antibacterial agents, remdesivir, and lopinavir/ritonavir. Ischemia due to vascular coagulopathy, thrombosis, and systematic inflammatory cytokines storm are other contributing factors to significant liver damage in patients with severe COVID-19 infection [15,172].

## 4. Mechanisms of SARS-CoV-2 Infection in the Pancreas

To date, there is insufficient data to establish a definitive causative mechanism of pancreatic injury related to COVID-19. There have been two prominent theories proposed describing the mechanisms of pancreatic damage seen in SARS-CoV-2 infection, including a direct and an indirect mechanism. The direct mechanism may be explained by studies showing that pancreatic acinar, ductal, and islet cells highly express ACE2 and TMPRSS2 receptors that promote SARS-CoV-2 cell entry [225,226,227]. A study conducted by Szlachcic et al. used in vitro models of human pancreatic cells to show that SARS-CoV-2 is capable of infecting and actively replicating in these cell types [226]. Furthermore, Liu et al. reported that in healthy individuals, the pancreas was found to express higher levels of ACE2 mRNA than in the lungs [225]. Indirect pancreatic injury may be induced by the systemic inflammatory and immune responses induced by SARS-CoV-2 and subsequent endothelial cell damage that mediates the leakage of these proinflammatory substances for distribution to various distant tissues, including the pancreas [228,229]. It is also possible that the virus may initially spread from the duodenal epithelium to ACE2-expressing acinar and islet cells via the pancreatic duct inducing a cytolytic effect that permits the release of pancreatic enzymes into the interstitial space. This may be mediated by infection-induced dysregulation of the gut microbiota and subsequent disruption of the intestinal barrier as described above.

Microscopic examination of COVID-19 pancreatic tissues at autopsy revealed the expression of entry receptors, severe fatty replacement of acinar cell mass, arteriosclerosis, and fibrosis [230,231,232,233]. During pancreatic inflammation, activated stellate cells release inflammatory cytokines and chemokines resulting in pancreatic fibrosis [234]. ACE2 downregulation caused by direct viral infection in pancreatic cells may disrupt ACE2/angiotensin-(1–7) interactions that normally regulate insulin resistance, insulin secretion, and pancreatic B cell survival [235]. Loss of ACE2 functions lead to accumulation of angiotensin II. Increased levels of angiotensin II dysregulates the protective arm of the RAAS, resulting in increased fibrosis, inflammation, and hypertension [236]. Multiple cases of acute pancreatitis in patients with COVID-19 have been reported in the literature [36,237,238,239,240] and altered serum cytokine levels of increased IL-6, IL-8, and IL-10 were found to be similar in cases of severe acute pancreatitis and in cases of COVID-19 [229]. Higher IL-6 levels are a recognized severity marker for both diseases individually [241,242]. A case-series study of 52 COVID-19 patients showed elevated amylase and lipase levels in 17% of the cases [243]. Previous studies reported that leakage of pancreatic triglyceride lipase in visceral adipose tissue, including intrapancreatic adipose tissue, results in increased adipose tissue lipolysis which generates excess nonesterified fatty acids [229,244]. These toxic fatty acids are known to inhibit mitochondrial complexes I and V, and cause pancreatic necrosis, ARDS, and multisystem organ failure [245]. Thus, acute pancreatitis in COVID-19 patients may potentially occur directly due to the cytopathic effect of SARS-CoV-2, secondary to leakage of pancreatic intracellular enzymes that result in autodigestion and cell death, or indirectly by cytotoxic immune responses.

## 5. Conclusions

While COVID-19 is mainly an upper and lower respiratory disease, increasing cases and reports point to the involvement of extrapulmonary organs and systems by SARS-CoV-2 infection. Gastrointestinal symptoms are increasingly reported in patients with COVID-19, and studies of these patients revealed the presence of the virus in enterocytes, pancreatic epithelial and islet cells, hepatocytes, and cholangiocytes. While the abundance of ACE2 receptors on these cells makes them highly susceptible to SARS-CoV-2 infection, the autophagic, vesicular, and metabolically active machinery creates an ideal microenvironment for viral replication, proliferation, and systemic transmission. The mechanisms of gastrointestinal injuries are largely multifactorial and involve a myriad of alterations to autophagy, immune evasion, and hyperinflammatory responses that contribute to GI symptomology and may compound to create a series of catastrophic events that can progress to an unsustainable disease state. Elevated transaminase and pancreatic enzyme levels following severe COVID-19 infections may be associated with viremia-induced systemic inflammatory response syndrome that can lead to sepsis. Additionally, elevated liver enzymes in severe cases of COVID-19 may indicate the possibility of hepatic and pancreatic damage that further contribute to the severity of the disease. It is critical to monitor these enzymes as they may predict a poorer prognosis. Viral shedding in the stool points to a possible fecal-oral transmission route, so it is essential to implement precautionary measures to prevent future re-infections. Hematologic studies should be conducted regularly in patients with coagulative risk factors, more severe disease states, and immobile patients, as viral presence in the blood has been implicated in alterations involving coagulation cascades, the development of thrombi and emboli, and systemic inflammatory-related burdens. Further studies are needed to determine potential treatments that may inhibit and mitigate the effects of autophagic and immune dysfunction.

## Data Availability

Not applicable.
